# Fungi and mycotoxins during conventional and improved postharvest processing and storage of local rice in Nigeria

**DOI:** 10.1007/s12550-026-00683-9

**Published:** 2026-10-06

**Authors:** Oluwawapelumi A. Oyedele, Muiz O. Akinyemi, Michael Sulyok, Rudolf Krska, Jos Houbraken, Chibundu N. Ezekiel

**Affiliations:** 1 Department of Microbiology, Babcock University, Ilishan Remo, Ogun State Nigeria; 2 Westerdijk Fungal Biodiversity Institute, Uppsalalaan, 3584 CT Utrecht, The Netherlands; 3 Unit for Environmental Science and Management, Potchefstroom Campus, North-West University, Potchefstroom, 2531 Republic of South Africa; 4 Department of Agricultural Sciences, Institute of Bioanalytics and Agro-Metabolomics, BOKU University, Konrad Lorenz Str. 20, Tulln, 3430 Austria; 5 Institute for Global Food Security, School of Biological Sciences, Queen’s University Belfast, University Road, Belfast, Northern Ireland BT7 1NN UK

**Keywords:** Aflatoxin, Food processing, Food safety, Hermetic storage, Mould, Secondary metabolites

## Abstract

This study applied a polyphasic characterization approach and LC-MS/MS analysis to elucidate fungal and mycotoxin contamination dynamics during conventional (CPRP) and improved (IPRP) postharvest processing and a 90-day storage of rice in Nigeria. Three hundred and twenty-five fungal isolates belonging to 21 genera (10 moulds, one crust fungus, 10 yeasts) and 54 species were recovered from rice across both processing chains. The CPRP chain had greater diversity of fungal genera compared to the IPRP chain. *Aspergillus* predominated both processing chains while yeasts occurred only in the CPRP chain. Species belonging to *Aspergillus* sections *Aspergillus*, *Candidi*, *Flavi* and *Nidulantes*, and *Penicillium* section *Citrina* predominated in stored rice in the two processing chains. A total of 106 extrolites including several uncommon extrolites and potential species markers were identified in agar cultures of 38 strains from 19 mould species. Fifteen mycotoxins were detected in rice along the processing and storage chains. Processing of rice in the IPRP chain and hermetic storage for 90 days reduced total aflatoxins by 9% compared to non-reduction in CPRP chain. Conventionally processed and stored rice in jute bags after 90 days recorded at least twice higher percentage reductions of total fumonisin, nivalenol and tenuazonic acid compared to hermetically stored rice from the IPRP chain. However, citrinin+dihydrocitrinone levels in rice from the two storage types increased progressively during the 90-day storage period. This study highlights the critical control points in the two rice process chains and provides insights into possible mycotoxin control approaches along the chain including in storage.

## Introduction

Rice (*Oryza sativa* L.) is the third most produced cereal globally, with a total worldwide production of 520.4 million metric tonnes in 2023/2024 (Shahbandeh 2024; USDA FAS 2024). The 2023 annual ranking of highest rice producing countries placed Nigeria as 14th globally and first in Africa, with an estimated 5.36 million metric tonnes produced (USDA FAS 2024). Rice contributes approximately 21% of the global per capita caloric intake (Awika 2011). Nigeria ranks the 10th highest global consumer of rice, with 7,700 thousand metric tonnes for 2023 and per capita consumption of 32 kg, emphasizing its importance as one of the staple foods consumed across different ethnic groups and by all ages (PwC 2018). Consequently, rice is considered a major dietary contributor to global food security. The utility of rice in human nutrition includes household food and complementary foods for infants and young children (Ezekiel et al. 2019; Ojuri et al. 2018). In animal nutrition, rice is used as fodder for ruminants and feed ingredients in pet food (Aquino et al. 2020), while its application in the industry includes its use as raw material in producing sweeteners and concrete in building construction (Khan et al. 2022; Khedheyer Al-Alwan et al. 2024).

Similar to many other countries in sub-Saharan Africa (SSA), rice production in Nigeria is largely undertaken by subsistence farmers, and the crop is grown in nearly 85% of all states (Ayanlade and Radeny 2020). Nonetheless, the quantities produced in-country are insufficient to cater for the increasing consumption of this staple; consequently, large tonnes of rice are imported into the country annually to supplement local production, despite the numerous efforts towards its improvement (Ismaila et al. 2013). Once rice is harvested, postharvest processing and storage are paramount to avoid crop loss, ensure food security, and preserve seeds for planting in the next season. Generally, harvested and threshed rice, known as paddy due to the covering of grains by the husks (Gonçalves et al. 2019), is separated from the husk in a winnowing process to release the grains. During the winnowing process, grains are soaked and parboiled to enhance easy removal of husks; the duration of the two intermediate steps may vary depending on the processor. Winnowed rice is then dried, milled and stored.

The quality of grain is strongly influenced by processing, storage infrastructures, and handling practices. In subsistence farmer settings across many developing countries, including Nigeria, sun-drying of grains in open environments, often without an underlay, and grain storage in conventional jute bags under poorly aerated environments are common practice (Ayeni et al. 2021). These conditions create favourable environments for the growth of (toxigenic) moulds and subsequent mycotoxin contamination, predisposing consumers to health risks, economic and nutritional losses (Ayeni et al. 2021; Makun et al. 2011a). To ensure optimal drying and storage of grains under conditions that minimize insect infestation and fungal growth, retain grain quality, and increase food security, solar and mechanical dryers (Gonçalves et al. 2019) as well as improved/hermetic storage silos and bags such as GrainPro and the Purdue Improved Crop Storage (PICS) bags, have been introduced in developing countries. Hermetic storage facilities function effectively by creating an oxygen-depleted, carbon IV oxide (CO_2_)-enriched microenvironment that limits aerobic life (insects and microorganisms) through asphyxiation and desiccation (Navarro et al. 1994). Studies have shown hermetic storage is effective for optimal and prolonged storage of different crops including maize, peanuts, and wheat (Diarra and Amoah 2019; Kalsa et al. 2019; Sudini et al. 2015; Williams et al. 2017a, b). Nevertheless, data on the application of these hermetic technologies in rice storage are sparse (Baoua et al. 2016; Covele et al. 2020; Hossain et al. 2019). Furthermore, hermetic storage of grains in the context of mycotoxin control focused mainly on aflatoxins (Diarra and Amoah 2019; Murashiki et al. 2018; Ng’ang’a et al. 2016; Sudini et al. 2015; Walker et al. 2018; Williams et al. 2014), with limited data on fumonisins (Murashiki et al. 2018; Walker et al. 2018). None of the hermetic storage studies on rice considered mycotoxins.

Pest invasion is a major threat to efficient rice production from farm to the consumer’s table, especially contamination by toxigenic fungi that produce mycotoxins in the field and, more importantly, during postharvest handling and storage under poor conditions (Makun et al. 2011b; Tang et al. 2019). *Aspergillus*, *Fusarium* and *Penicillium* species are commonly associated with rice (Abdus-Salaam et al. 2016; Ekpakpale et al. 2021; Ezekiel et al. 2020a; Makun et al. 2007; Qi et al. 2022; Reddy et al. 2008), and aflatoxins, deoxynivalenol (DON), fumonisins, ochratoxin A (OTA), and zearalenone (ZEN) were the most frequently reported mycotoxins (Abdus-Salaam et al. 2015; Ekpakpale et al. 2021; Eslamizad et al. 2021; Ezekiel et al. 2021; Mahdjoubi et al. 2020; Onyedum et al. 2020; Silva et al. 2023; Sinphithakkul et al. 2019; Siri-Anusornsak et al. 2022). The aforementioned studies focused on identifying moulds, quantifying regulated mycotoxins or investigating both contaminants. However, many of the aforementioned reports investigated processed or stored rice from local markets, with sparse data on contamination during the postharvest processing stages (Monteiro et al. 2025; Tang et al. 2019). In fact, a review of global mycotoxin occurrence in rice recently reported 50% of polished rice in African stores was contaminated, and the highest aflatoxin level found was 1,642 µg/kg, which was twice as high as the level in maize from Asia (Lee and Ryu 2017).

Despite the foregone, data on secondary metabolite production from rice-associated strains in Nigeria and elsewhere remain limited. Secondary metabolite profiling of fungal strains is important to: (a) determine other possible compounds asides regulated mycotoxins that can be produced in rice due to potential synergistic effects; (b) complement fungal classical and molecular identification data; (c) document the spectrum of small compounds of public health and industrial applications; and (d) provide chemical data relevant to facilitate strain exploitation for other uses (Frisvad et al. 2007a, 2018; Samson et al. 2019). In view of the growing need to improve local rice production through optimizing current and implementing new postharvest processing and storage practices, it is imperative to elucidate the fungal and chemical contaminants profiles during rice processing and storage. This study aimed to gain insight in the fungal growth and mycotoxin contamination in rice during conventional and improved postharvest processing and storage methods practiced by the local farmers in Nigeria. The goal was to prevent crop loss and increase food security through the prevention of spoilage postharvest and mycotoxin control in rice.

## Materials and methods

### Study design and sampling

**Fig. 1:**
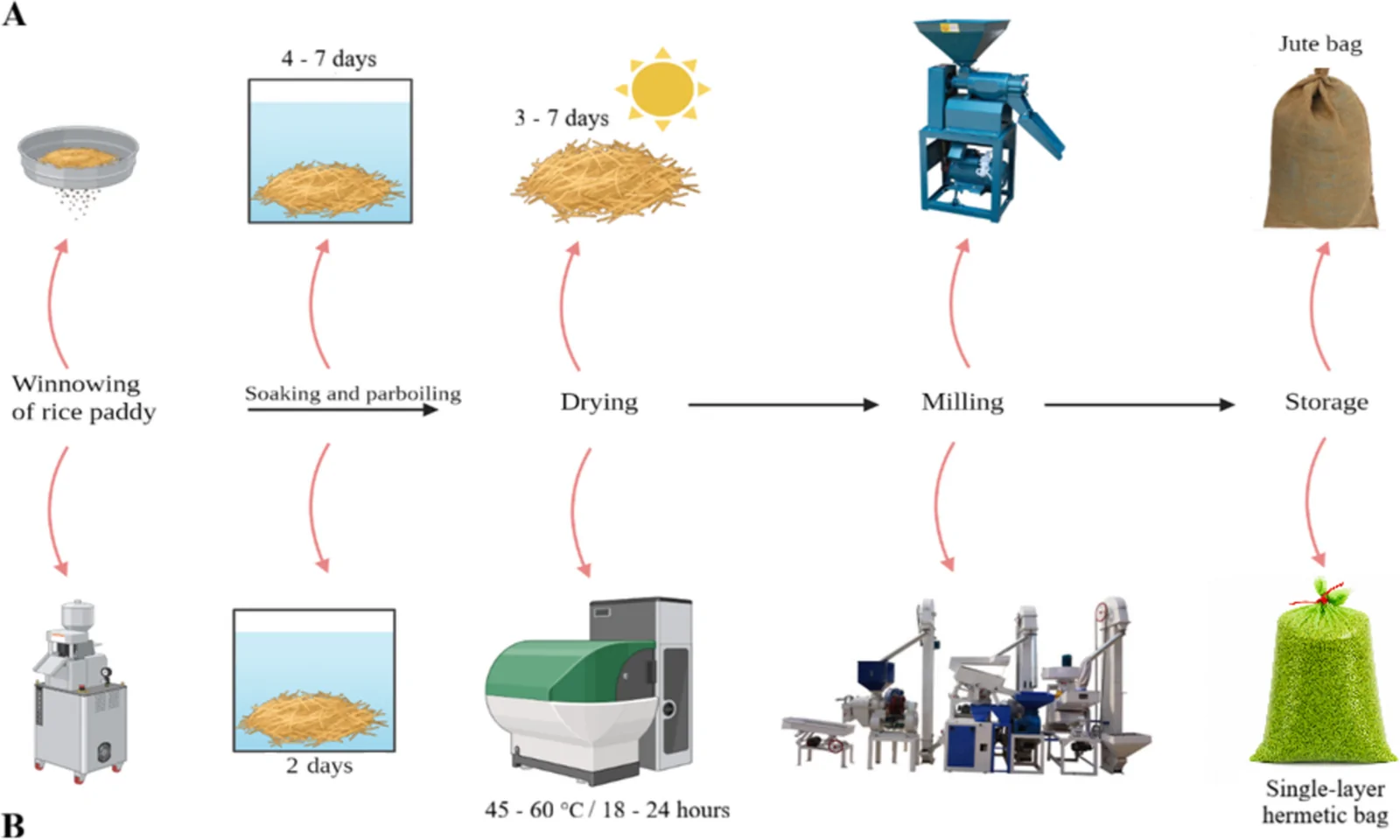
Schematic diagram of the (**A**) conventional and (**B**) improved postharvest rice processing and storage chains

An experimental study was conducted in two states (Ekiti and Ogun) in southwest Nigeria. Ekiti and Ogun states represent two of Nigeria’s top 10 rice-producing states (Commodity Port 2022), and they were selected to reflect different postharvest rice processing practices (conventional [CPRP] and improved [IPRP]) investigated in this study. A total of 27 rice farmers were purposely selected and recruited into the study; 13 in Ogun state and 14 within Ekiti state. The criteria for inclusion in the study included: active cultivation of rice (at least one hectare of farm), adoption of a specified postharvest rice processing practice, involvement in the local rice farmers’ cooperative association, and willingness to allow sampling at prespecified intervals. Farmers in Ogun state adopted CPRP practices (soaking of manually-winnowed rice paddy in tap water at ambient temperature for 4–7 days prior to parboiling at 95–100 °C for 5 h, sun drying on concrete slabs for 3–7 days, milling, and storage of dried rice in jute bags), whereas those in Ekiti state implemented IPRP practices (soaking of manually-winnowed rice paddy in hot water (up to 65 °C) for 2 days prior to parboiling at 95–100 °C for 30 min, drying in a mechanical drier at 45–60 °C for 18–24 h, milling, and storage of dried rice in single-layer hermetic-type bags) (Fig. 1). Single-layer hermetic-type bags were selected for cost-effectiveness at the farmer household level. Storage of dried rice in the hermetic-type bags was an intervention step introduced in this study to complete the IPRP practices since farmers generally use conventional jute bags. The duration of rice storage was 90 days at ambient temperature.

Winnowed, dried, and milled rice samples were collected at the farmer’s location. At each sample point, one bulk sample was collected per rice farmer resulting in 81 rice samples (Ogun state = 39, 13 farms; Ekiti state = 42, 14 farms). Each bulk rice sample weighed 10 kg each and was collected from 10 random rice bags, containers or heaps using a surface-sterilized probe for the bags or trowel for open container vessels or heaps. The 10 random rice bags, containers or heaps were selected by sampling one bag, container or heap per every 10 bags, containers or heaps. To obtain the bulk sample, 1 kg sub-sample was taken from three parts (top, middle and bottom) of each bag, container or heap until 10 kg was achieved. For the milled rice, four times as much as the sample size was collected due to partitioning of the samples into 30, 60 and 90 storage durations. For the storage experiments, the milled rice, which served as time zero of storage, was utilized. Each milled rice was divided into the appropriate bags and storage time. Storage bags were unopened until their time of analysis/end of storage duration. Each sample collected from the rice farmers served as replicates for the study.

For laboratory analysis, 1 kg sub-sample was taken representatively from a 10-kg bulk sample for one farmer or storage duration set-up and subjected to analysis. All samples were collected into clean labelled polyethylene bags and transported to the laboratory for further analysis. Each 1 kg sample for analysis was subdivided into two equal parts: batch A for moisture analysis and mycological analysis, and batch B for mycotoxin analysis. Samples belonging to batch A were stored at 4 °C prior to analysis within 24 h, whereas batch B samples were stored at −20 °C for mycotoxin analysis. Prior to further appropriate analysis, 1 kg sample of each batch was grinded into fine powder using an electric blender (MX-AC400, Panasonic, India).

### Moisture content analysis of rice

Moisture content analysis was performed on the rice samples by employing the oven-drying to constant weight method according to prescriptions by EC (2000). The heating temperature of a UNISCOPE hot air oven (SM9053, Surgifriend Medicals, England) was set at 105 °C and samples were weighed every hour until constant weight was achieved. Triplicate readings were taken per rice sample.

### Mycological analysis of rice

#### Isolation and enumeration

The dilution plating technique as described by Samson et al. (2019) was employed to isolate and enumerate fungi in the rice samples. Briefly, 10 g of each ground rice sample was diluted in 90 mL of sterile distilled water and homogenised for 2 min on a vortex mixer. Thereafter, 0.1 mL of the suspension was plated out on the agar media dichloran 18% glycerol agar (DG18) and malt extract agar (MEA). The plates were incubated at 25 °C for 7–10 days. Afterwards, all fungal colonies were counted and recorded as colony forming units per gram (CFU/g). The CFU/g data were then logarithm transformed at Log_10_ in order to create a normal distribution. Distinct fungal colonies on the isolation plates were transferred to freshly prepared DG18 and/or MEA plates and incubated at 25 °C for 7 days. Afterwards, pure cultures were preserved on MEA slants in 4 mL vials overlaid with sterile distilled water.

#### Characterization of fungi

The diversity of fungi recovered from the rice samples were characterized by adopting the polyphasic approach as described by Ezekiel et al. 2020a, b). Characterization of moulds involved macro- and micro-morphological character examination, sequenced-based typing and secondary metabolite screening of fungal cultures, whereas yeasts were identified by only pheno- and genotyping methods. Briefly, the isolates were three-point inoculated on MEA and subsequently incubated at 25 °C for 7 days. Thereafter, the cultures were assessed for macroscopic and microscopic characters and the features compared with descriptions in appropriate keys (Frisvad and Samson 2004; Leslie et al. 2006; Pitt and Hocking 2009; Samson et al. 2011a, 2019).

Prior to molecular typing, a selection of phenotypically characterized isolates based on dominance on isolation plates and representatives of all phenotype groups were made. The selected phenotyped isolates were then subjected to molecular typing. Genomic DNA of 5-days old cultures on MEA were extracted with the DNeasy Ultraclean Microbial kit (Qiagen). A part of the β-tubulin (*BenA*) and/or calmodulin (*CaM*) gene was amplified and thereafter sequenced for all *Aspergillus*, *Penicillium*, and *Talaromyces* isolates (Houbraken et al. 2011, 2012; Samson et al. 2019), while the ITS regions, a part of the translation elongation factor 1 alpha (TEF-1α), and/or a part of the RNA polymerase II subunit (RPB2) gene were amplified and sequenced for other genera (Chen et al. 2015, 2017; Groenewald et al. 2005, 2013). The generated sequences were compared with those present in the National Center for Biotechnology Information (NCBI) database (GenBank) and the curated database housed at the Westerdijk Fungal Biodiversity Institute (WI). The identified isolates were deposited and are maintained in the DTO working culture of the WI. All the generated sequences can be found in the GenBank under accession number PX741427-PX741495.

Further characterization of the moulds was performed by profiling secondary metabolites produced in culture of selected isolates using a targeted LC-MS/MS method. Isolate selection was based on representativeness of the various sections of occurring moulds. To achieve this, the isolates were grown on Czapek yeast autolysate (CYA) agar, yeast extract sucrose (YES) agar, MEA and oatmeal agar (OA) as appropriate for the genera or species, and then incubated 25 °C for 7–21 days (Samson et al. 2019). Thereafter, agar plugs of each culture were extracted using ethylacetate/dichloromethane/methanol (3:2:1, v/v/v) as described by Filtenborg et al. (1983). The extracts were subjected to LC-MS/MS analysis for secondary metabolite screening as outlined below.

### Multi-metabolite analysis of fungal culture extracts and rice

Secondary metabolites including mycotoxins present in the extracts of fungal cultures and sampled rice along the two postharvest processing chains were determined using a dilute and shoot LC-MS/MS method described by Sulyok et al. (2020). Dried fungal culture extracts were dissolved in 1 mL (ratio 1:1, v/v) of extraction solvent (acetonitrile/water/acetic acid 79:20:1, v/v/v) and then diluted (1:1, v/v) with acetonitrile/water/acetic acid 20:79:1, v/v/v prior to injection into the LC-MS/MS instrument. For the rice samples, 5 g from the homogenised ground samples were mixed with 20 mL of extraction solvent in a 50 mL polypropylene tube (Sarstedt, Nümbrecht, Germany). Exactly 0.25 g of the food samples were spiked with a multi-analyte stock solution to determine the apparent recovery of the method. The spiked samples were stored overnight in order to reach equilibrium, then mixed with 1 mL of extraction solvent. The extraction of rice and spike samples were performed on a GFL 3017 rotary shaker (GFL, Burgwedel, Germany) for 90 min. Afterwards, 500 µL of each extract was diluted 1:1 (v/v) with dilution solvent prior to injection into the LC-MS/MS instrument for measurement.

Metabolites were screened on a QTrap 5500 LC-MS/MS System (Applied Biosystem, Foster City, CA, United States) equipped with TurboIonSpray electrospray ionisation (ESI) source and a 1290 Series HPLC System (Agilent, Waldbronn, Germany). Chromatographic separation was performed at 25 °C on a Gemini^®^ C18-column, 150 × 4.6 mm i.d., 5 μm particle size, equipped with a C18 4 × 3 mm i.d. security guard cartridge (Phenomenex, Torrance, CA, United States). The chromatographic method, chromatographic and mass spectrometric parameters were as described by Sulyok et al. (2020). ESI-MS/MS was conducted in the time-scheduled multiple reaction monitoring (MRM) mode both in positive and negative polarities in two separate chromatographic runs per sample by scanning two fragmentation reactions per analyte. The MRM detection window of each analyte was set to its expected retention time ± 20 s and ± 26 s in the positive and the negative modes, respectively. The identified positive analytes were confirmed by the acquisition of two MRMs per analyte (with the exception of moniliformin (MON), which exhibited only one fragment ion). This yielded 4.0 identification points according to European Commission decision 2002/657 (EC 2002). In addition, the LC retention time and the intensity ratio of the two MRM transitions agreed with the related values of an authentic standard within 0.03 min and 30% respectively.

Metabolites were quantified based on linear, 1/x weighed calibration using serial dilutions of the external multi-component stock solution. Calculated concentrations were corrected for apparent recoveries. Limits of detection (LOD) and limits of quantification (LOQ) were determined based on the standard deviation of samples spiked at low concentration levels following the EURACHEM guide (Magnusson and Örnemark 2014). A general expanded measurement uncertainty of 50% (coverage factor k = 2) was previously determined for the method (Stadler et al. 2018). Quality control measures adopted are as described in Ezekiel et al. (2024).

### Data analysis

All the data collected, excluding the dataset obtained from molecular analysis, were analysed using R software version 4.5 utilizing packages including lme4, lmerTest, and ggplot2 for analytical and graphical outputs. Descriptive statistics were calculated on fungal count and moisture content data as means with standard errors. Pearson correlation analysis quantified the linear association between moisture content and log_10_-transformed colony forming units per gram (CFU/g) sample. Linear regression modelling examined predictive relationships through the equation: CFU ~ Moisture * Treatment (conventional or improved), incorporating both main effects and their interaction. A three-way factorial ANOVA (CFU ~ Moisture * Treatment * Processing Stage) assessed simultaneous effects of experimental factors. Finally, a linear mixed effects model (CFU ~ Moisture * Treatment + [1/Processing Stage]) accounted for potential non-independence within rice type categories. Statistical significance was determined at α = 0.05, with exact p-values reported to three decimal places. Effect size measures included correlation coefficients (r), coefficients of determination (R²), and variance components from mixed models.

## Results and discussion

### Moisture content of rice along the process chains

**Fig. 2:**
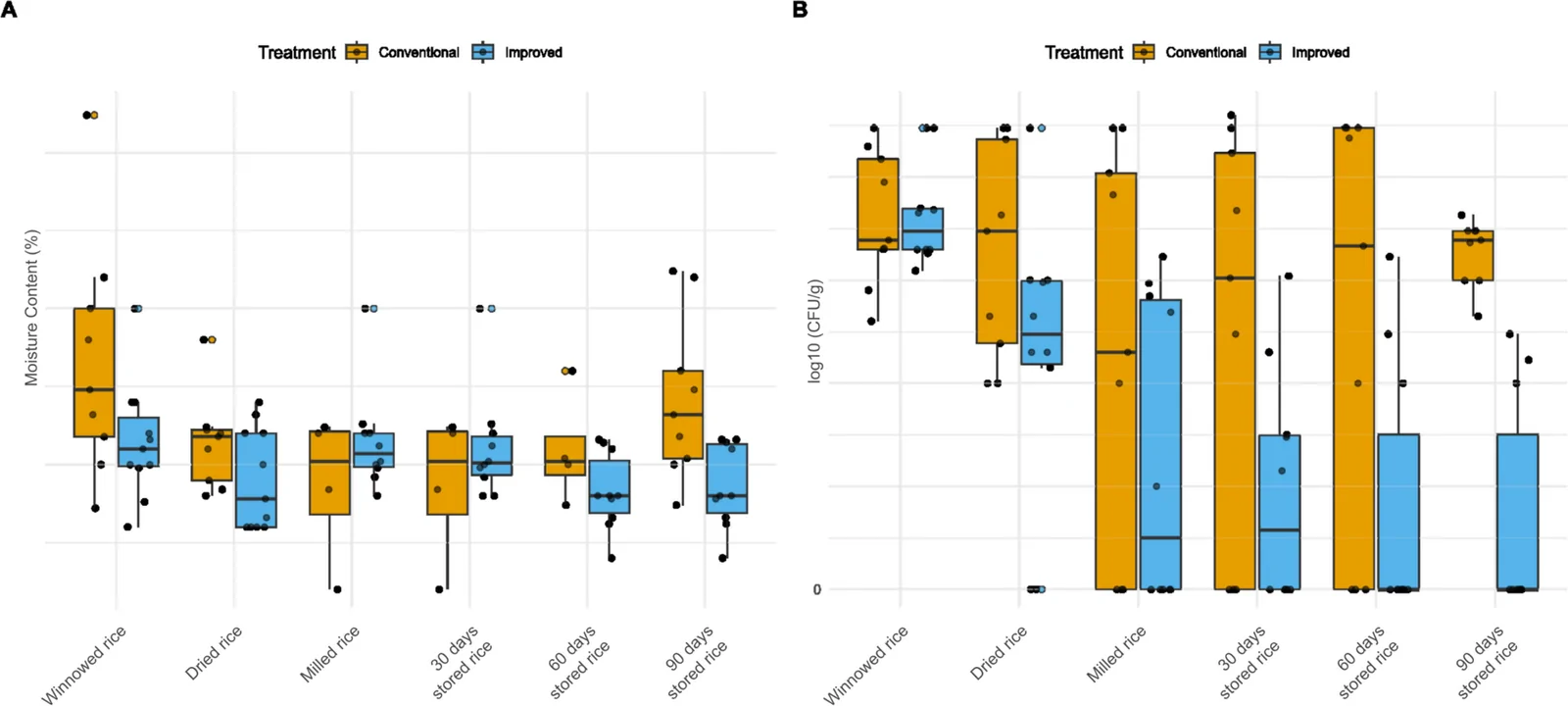
(**A**) Moisture content and (**B**) colony forming unit per gram of rice sampled across the conventional and improved postharvest processing and storage chains

Moisture level is a critical determinant of the extent of fungal and mycotoxin contamination of grains (Ng’ang’a et al. 2016; Walker et al. 2018; Williams et al. 2017a). Thus, it is recommended to keep moisture levels of grains, including rice, at below 14% for safe storage (Magan and Aldred 2007; Ramli et al. 2021). In the present study, the moisture contents of rice samples at each stage of postharvest rice processing from winnowing to 60 days in storage were variable and are depicted in Fig. 2A. Generally, the mean moisture levels were higher in rice from the CPRP chain (winnowed: 16.7 ± 7.3%; dried: 11.6 ± 2.5%; milled: 10.0 ± 4.2%; 30 days stored: 8.7 ± 4.8%; 60 days stored: 11.0 ± 3.6%; 90 days stored: 13.5 ± 3.5%) compared to the IPRP chain (winnowed: 11.8 ± 3.5%; dried: 9.5 ± 3.0%; milled: 10.0 ± 5.6%; 30 days stored: 10.7 ± 3.3%; 60 days stored: 8.5 ± 2.2%; 90 days stored: 8.9 ± 2.4%), albeit no significant difference (*p* > 0.05) between the means was recorded at each stage (Fig. 2A).

Despite the aforementioned observation in the estimated means, rice processed via the IPRP chain consistently had lower moisture levels below the recommended 14% maximum moisture limit for safe storage of rice. While storage in the conventional jute bags progressively increased moisture contents of the rice grains all through the 90 days of storage, a contrasting decline and then static trend was recorded for moisture contents in rice under storage in single-layer hermetic-type bags. In the unmodified environment, moisture content of grains fluctuates with changes in ambient temperature and relative humidity (Mlambo et al. 2017); this may have accounted for the constant moisture content increase in rice grains stored in conventional jute bags. Conversely, storage of rice in single-layer hermetic-type bag limited the absorption of environmental moisture resulting in an initial drop and stabilization of its level in the micro-environment; this agrees with similar reports for maize (Mlambo et al. 2017; Walker et al. 2018; Williams et al. 2017a) during their storage in hermetic devices.

### Distribution of fungi in rice along the conventional and improved postharvest processing chains

#### Fungal load and incidence

Quantitative microbiological growth and survival data are widely utilized parameters to determine food quality in microbiology (Jay 1998). The typical expected range of fungal load in cereal grains in commercial channels is 2–4 Log (CFU/g), beyond which is considered high (Minutillo et al. 2022). The mean fungal loads (Log_10_CFU/g) in rice processed through the CPRP chain at winnowing, drying, milling, 30 days storage, 60 days storage and 90 days storage stages were 4.0 ± 0.7, 3.6 ± 1.1, 2.3 ± 1.8, 2.6 ± 2.0, 2.6 ± 1.9 and 3.4 ± 1.1 while the mean fungal loads for the IPRP chain were4.0 ± 0.5, 2.1 ± 1.2, 1.4 ± 1.5, 1.0 ± 1.3, 0.8 ± 1.3 and 0.7 ± 1.2, respectively (Fig. 2B). The recorded data indicate that rice from both process chains had fungal load within the recommended limits earlier specified. However, there was drastic reduction in fungal load of rice in the IPRP chain from the winnowing stage to the end of the 90 days storage compared to the trend for the rice in the CPRP chain. Pearson correlation revealed a highly significant (*p* < 0.05) moderate positive correlation (*r* = 0.33) between changes in moisture content at each processing stages and CFU. A mixed effects model, accounting for variability between processing stage as a random effect, revealed that the improved treatment significantly reduced CFU levels (β = −1.65, *p* < 0.05).

The reduction in fungal load in rice of both process chains from winnowing to milling may be due to the floatation washing, drying and milling steps. First, rice processing involves floatation washing during and after soaking; this step that has been effective in mycotoxin reduction during maize processing may have led to removal of highly infected rice paddy and grains prior to drying of the remaining grains (Matumba et al. 2015). Thereafter, rice from the IPRP chain were constantly exposed to elevated temperatures of 45–60 °C in a mechanical drier for prolonged hours (18–24 h) prior to cooling and storage in hermetic bags compared to rice from the CPRP chain which were sun-dried slowly on slabs at ambient temperature that peaked at 34–36 °C for 4–5 h daily until assumed dry enough (approximately 3–7 days depending on the intensity of sunlight). Drying and milling of grains are usually performed under high temperature and can generate heat leading to low fungal counts; thus, the higher temperature regime for the two steps may have influenced the lower fungal load in rice from the IPRP chain. The general pattern observed for the fungal load in the two chains seem to be mediated by the moisture levels, which agrees with the fact that postharvest fungal growth in food is largely influenced by moisture levels (Magan and Aldred 2007; Ng’ang’a et al. 2016). The further reduction in fungal load in rice of the IPRP process chain from milling to the end of storage in contrast to the increase recorded in the CPRP chain is attributed to rice storage in the single-layer hermetic-type bags. The hermetic bags modified the storage internal environment by preventing atmospheric gases/oxygen exchange with the environment such that a low oxygen-high CO_2_ tension was created leading to a reduction in the number of viable fungal propagules within the rice (Diarra and Amoah 2019; Sudini et al. 2015; Walker et al. 2018). Specifically, high CO_2_ limits growth of moulds due to their aerobic metabolic nature (Moseley et al. 1971).

#### Genera and species diversity

**Fig. 3:**
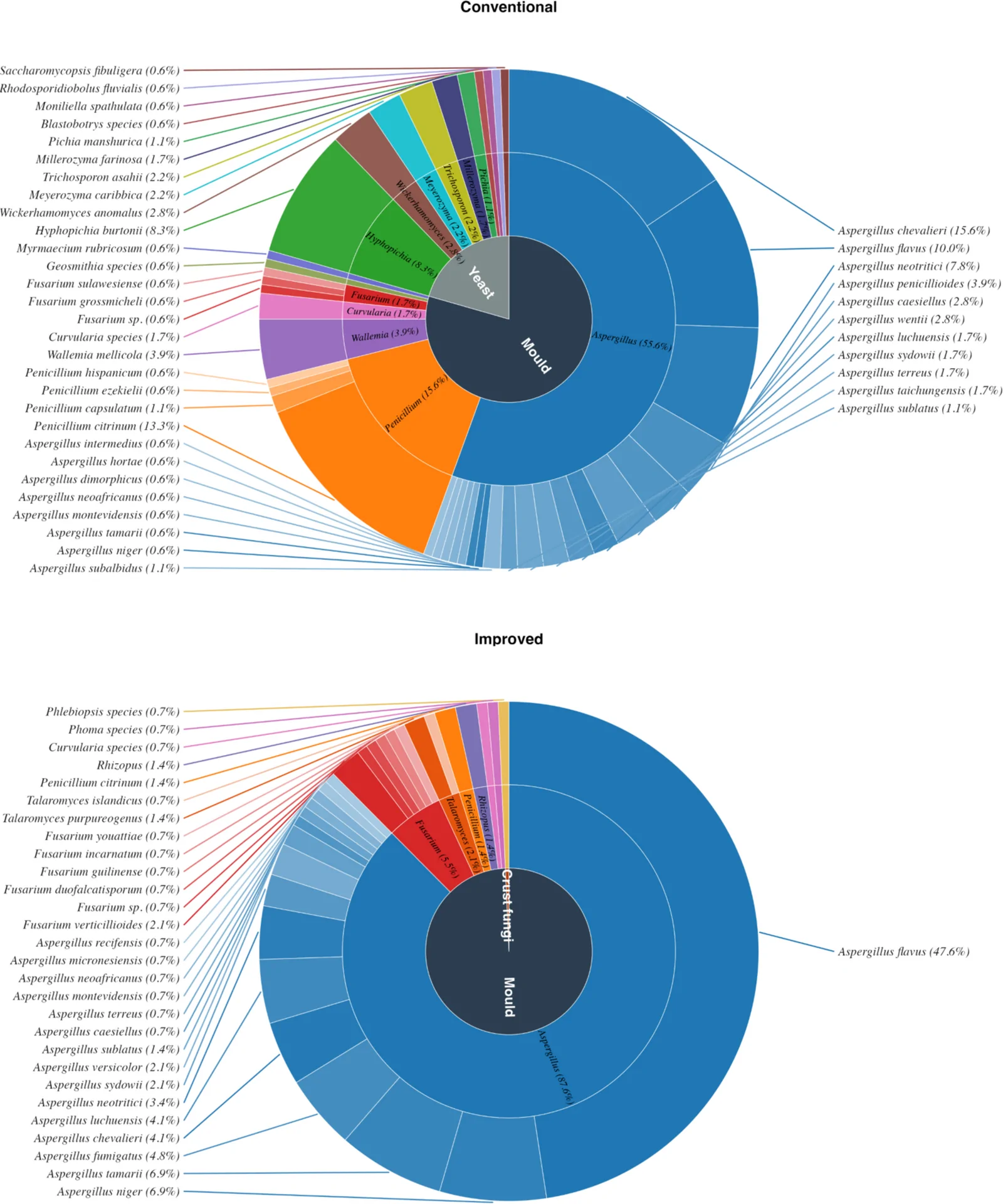
Incidence of fungal genera and species (percentage) in rice sampled along the conventional and postharvest processing chains

Rice samples from the two process chains yielded 325 fungal isolates: 180 and 145 from the CPRP and IPRP chains, respectively. The isolates belonged to three fungal groups: moulds (88,3%, crust fungi (0.3%) and yeasts (11.4%). The fungal community in rice from the CPRP chain consisted of moulds and yeasts (Fig. 3A), whereas moulds and crust fungi were present in the IPRP chain (Fig. 3B). In the CPRP chain, the incidence of moulds (79.4%) was higher than the incidence of yeasts (20.6%); nevertheless, the yeasts were more diverse with 10 genera compared to moulds which comprised seven genera (Fig. 3A). This observation of yeasts occurring only in the CPRP chain may have been due to the ambient tap water-soaking for prolonged duration (4–7 days) compared to the shorter duration of 2 days hot water-soaking in the IPRP chain. The type of soaking water (cold vs. hot) adopted was documented to influence the microbial population in rice processing, with higher yeast populations when soaking with cold water was employed (Ramalingam and Anthoni Raj 1996). In addition, the mechanical drying of rice at elevated temperatures reaching 60 °C in the IPRP process chain was more effective to control fungal (yeast, mould and crust fungi) loads compared to sun-drying adopted in the CPRP. Furthermore, the predominance of yeasts in the later stages of fermentation of foods, including rice, was previously reported (Adeniran et al. 2012; Ramos et al. 2010).

The incidences of fungal genera in rice from the CPRP chain in descending order of magnitude were *Aspergillus* (55.6%), *Penicillium* (15.6%), *Hyphopichia* (8.3%), *Wallemia* (3.9%), *Wickerhamomyces* (2.8%), *Meyerozyma* (2.2%), *Trichosporon* (2.2%), *Fusarium* (1.7%), *Millerozyma* (1.7%), *Pichia* (1.1%), *Blastobotrys* (0.6%), *Curvularia* (1.7%), *Blastobotrys* (0.6%), *Geosmithia* (0.6%), *Moniliella* (0.6%), *Myrmaecium* (0.6%), *Rhodosporidiobolus* (0.6%), *Saccharomycopsis* (0.6%) (Fig. 3A). In the IPRP chain, *Aspergillus* (87.6%) also predominated over all other genera including *Fusarium* (5.5%), *Talaromyces* (2.1%), *Penicillium* (1.4%), *Rhizopus* (1.4%), *Curvularia* (0.7%), *Phlebiopsis* (0.7%), and *Phoma* (0.7%) (Fig. 3A). The predominance of *Aspergillus* in rice from both process chains and, additionally, *Penicillium/Talaromyces* and *Fusarium* from the CPRP and IPRP chains, respectively, agree with the reports of Abdus-Salaam et al. (2016), Gautam et al. (2012) and Makun et al. (2007). Precisely, Abdus-Salaam et al. (2016) reported *Aspergillus* and *Penicillium* as the predominant fungal genera/species in rice from Sudan Savannah (SS), Northern Guinea Savannah (NGS), Southern Guinea Savannah (SGS), Derived Savannah (DS) and Humid Forest (HF) agroecological zones in Nigeria. This predominance of *Aspergillus* and *Penicillium* may have been due to their xerophilic nature (Pitt 1975; Samson et al. 2019). Although eight *Fusarium* species were recovered in the samples from both chains, they are not considered to be xerophilic, and probably don’t grow during proper storage conditions (Samson et al. 2019). Moisture and water activity levels are critical to fungal growth and food spoilage (Pitt and Hocking 2022).

**Fig. 4:**
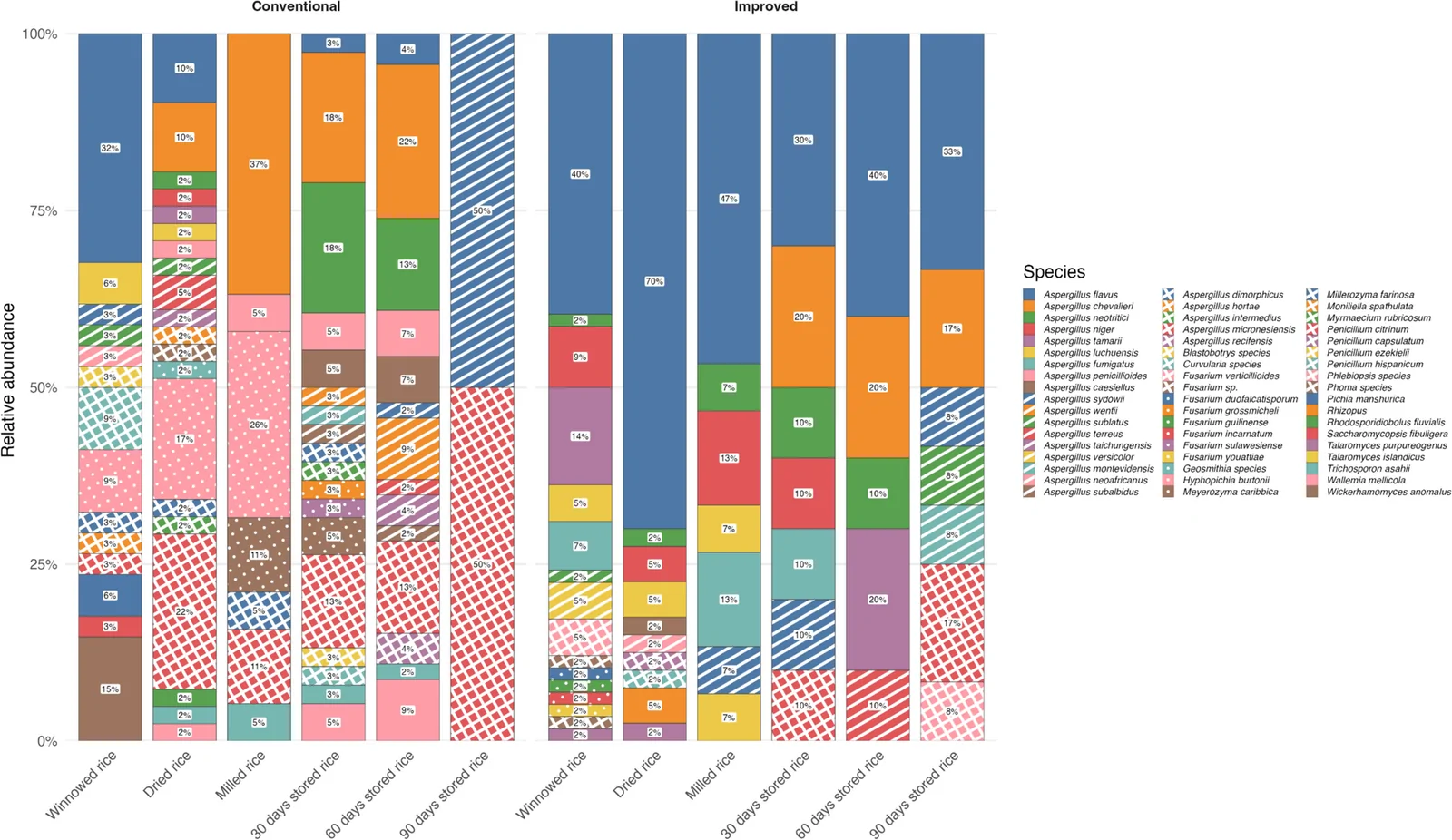
Incidence of fungal species in rice sampled across the conventional and improved postharvest processing and storage chains

The 21 fungal genera in the rice samples comprised 54 (43 moulds, 1 crust fungus and 10 yeasts) species (Fig. 4). Of the mould species recovered, those belonging to *Aspergillus*, *Penicillium* and *Talaromyces* were classified in 10, 3 and 2 sections, respectively (Table 1). Some species (e.g., *Aspergillus flavus*, *A. tamarii*, *A. niger*, *A. versicolor*, *A. wentii* and *P. citrinum*) within the identified sections are known mycotoxin producers (Chen et al. 2017; Ezekiel et al. 2020a, b; Houbraken et al. 2020; Sklenář et al. 2017; Samson et al. 2019). The species within sections *Aspergillus* (*Aspergillus chevalieri*, *A. intermedius* and *A. montevidensis*) and *Restricti* (*A. caesiellus* and *A. penicillioides*) initiate growth at very low moisture levels and increase the conduciveness of the environment for less xerophilic species including prolific mycotoxigenic species by increasing the water activity in the environment as a function of liberated metabolic water; this activity results ultimately in food spoilage (Houbraken et al. 2020; Kozakiewicz 1989; Semeniuk et al. 1947). Other notable xerophilic species found in the rice samples include *A. neotritici*, *A. wentii* and *Wallemia mellicola*; they adapt easily to osmotically challenged environments (Houbraken et al. 2020; Zajc et al. 2018; Zalar et al. 2005).

**Table 1:** Species and sections of selected moulds identified in rice processing chains in Nigeria

Fungal genera	Fungal sections	Fungal species	References
*Aspergillus*	*Aspergillus*	*Aspergillus chevalieri*, *A. intermedius*, *A. montevidensis*	Houbraken et al. 2020
	*Candidi*	*A. subalbidus*, *A. taichungensis*, *A. neotritici*	Glässnerová et al. 2022; Houbraken et al. 2020
	*Cremei*	*A. dimorphicus*, *A. wentii*	Houbraken et al. 2020
	*Flavi*	*A. flavus*, *A. tamarii*	Frisvad et al. 2019
	*Flavipedes*	*A. micronesiensis*	Sklenář et al. 2021
	*Fumgati*	*A. fumigatus*	Houbraken et al. 2020
	*Nidulantes*	*A. sydowii*, *A. versicolor*	Chen et al. 2016; Sklenář et al. 2022
	*Nigri*	*A. niger*, *A. luchuensis*	Visagie et al. 2024
	*Restricti*	*A. caesiellus*, *A. penicillioides*	Chen et al. 2017; Houbraken et al. 2020; Sklenář et al. 2017
	*Terrei*	*A. hortae*, *A. neoafricanus*, *A. terreus*, *A. recifensis*	Houbraken et al. 2020
*Penicillium*	*Cinnamopurpurea*	*Penicillium ezekielii*	Tan et al. 2022
	*Citrina*	*P. citrinum*	Visagie et al. 2014
	*Ramigena*	*P. capsulatum*, *P. hispanicum*	Visagie et al. 2014
*Talaromyces*	*Islandici*	*Talaromyces islandicus*	Houbraken et al. 2020
	*Talaromyces*	*T. purpureogenus*	Houbraken et al. 2020; Yilmaz et al. 2012

Data for species distribution in rice along the processing and storage stages are presented in Fig. 4. Several dominant species were respectively found at each stage of the CPRP and IPRP chains: *A. flavus* (32%), *Wickerhamomyces anomalus* (15%), and *A. flavus* (40%), *A. tamarii* (14%) for winnowed rice; *P. citrinum* (22%), *H. burtonii* (17%), and *A. flavus* (70%) for dried rice; *A. chevalieri* (37%), *H. burtonii* (26%), and *A. flavus* (47%), *A. fumigatus* (13%), *A. niger* (13%) for milled rice. In addition, dominant species in respective CPRP and IPRP storage chains were: *A. chevalieri* (18%), *A. neotritici* (18%), and *A. flavus* (30%), *A. chevalieri* (20%) for 30 days stored rice; *A. chevalieri* (22%), *A. neotritici* (13%), *P. citrinum* (13%), and *A. flavus* (40%), *A. chevalieri* (20%), *A. tamarii* (20%) for 60 days stored rice; and *A. sydowii* (50%), *P. citrinum* (50%), and *A. flavus* (33%), *A. chevalieri* (17%), *P. citrinum* (17%) for 90 days stored rice (Fig. 4). Besides the dominant species across the stages, certain species showed unique persistence patterns. For example, *A. flavus* persisted across all stages of the two processing and storage chains, whereas *A. neotritici* persisted across all stages of the IPRP and storage chains with incremental occurrence. *Aspergillus chevalieri*, *P. citrinum*, and *A. caesiellus* were respectively introduced during the drying stage of the CPRP and IPRP chains and persisted through to storage. *Aspergillus penicillioides* was introduced during milling and persisted into storage of rice processed via the CPRP chain. *Aspergillus montevidensis* was observed only during storage of rice from both processing chains. The diversity of species in the rice process chains, specific predominance and persistence of species classified in *Aspergillus* sections *Aspergillus*, *Candidi*, *Flavi* and *Penicillium* section *Citrina* across the processing chains and especially in stored rice emphasize the xerophilic nature of these species (Houbraken et al. 2020; Samson et al. 2019; Sklenář et al. 2017). Species in these sections play a critical role in spoilage of low moisture foods such as rice under storage due to their convenient growth at a_w_ of 0.7 compared to other species (Samson et al. 2019). In agreement with the present study, several (xerophilic) species were previously reported to be the predominant fungi in rice samples from field, market and farmers’ stores in Niger state (Makun et al. 2007, 2011b), stored rice from Himachal Pradesh, India (Gautam et al. 2012), and dried low moisture content read-to-eat foods in Nigeria (Ezekiel et al. 2020b). In addition, several species within the section *Aspergillus* including *A. chevalieri* and *A. montevidensis* have been documented in feedstuffs and dried fermented products (Greco et al. 2015; Hong et al. 2011; Pitt and Hocking 2009) while *A. neotritici* (section *Candidi*) has been reported in soil, food (grains and flour), indoor environments and outdoor air as a moderate xerophile (Glässnerová et al. 2022).

### Extrolites in cultures of moulds

Fungal extrolites are small molecule compounds produced as secondary metabolites into the environment of fungi, often as defensive, invasive and/or adaptation mechanisms. These compounds may include toxins, pharmaceutically relevant, industrial and biotechnologically important compounds (Frisvad and Samson 2004; Frisvad et al. 2007a, 2018). In mycology, chemotaxonomy is used to distinguish species based on the secondary metabolites they produce, and is complementary to fungal identification (Ezekiel et al. 2020b; Frisvad and Samson 2004; Frisvad et al. 2007a, 2018; Samson et al. 2019). Here, 106 extrolites were identified in agar cultures of 38 strains belonging to 19 mould (13 *Aspergillus*, 3 *Fusarium*, 2 *Penicillium* and 1 *Wallemia*) species recovered from the rice samples (Table 2). *Aspergillus flavus* and *A. versicolor* produced the most compounds (22 extrolites), while 19, 18, 18, 14, 13 and 11 compounds were found in cultures of *A. neoafricanus*, *A. niger*, *P. citrinum*, *A. caesiellus*, *A. chevalieri* and *A. subalbidus*, respectively. Other species in the present study produced less than 10 extrolites (Table 2).

**Table 2:** Extrolites in agar cultures of fungi isolated from rice

Extrolites	*Aspergillus caesiellus*	*Aspergillus penicillioides*	*Aspergillus chevalieri*	*Aspergillus montevidensis*	*Aspergillus flavus*	*Aspergillus tamarii*	*Aspergillus neoafricanus*	*Aspergillus niger*	*Aspergillus versicolor*	*Aspergillus subalbidus*	*Aspergillus taichungensi*s	*Aspergillus neotritci*	*Aspergillus wentii*	*Fusarium duofalcatisporum*	*Fusarium verticillioides*	*Fusarium youattiae*	*Penicillium citrinium*	*Penicillium ezekielii*	*Wallemia mellicol*a
3-Hydroxyterphenyllin	-	-	-	-	-	-	-	-	-	+	+	+	-	-	-	-	-	-	-
3-Nitropropionic acid	-	-	-	-	+/-	+	-	-	-	-	-	-	+	-	-	-	-	-	-
8-Acetylneosolanol	-	-	-	-	-	-	-	-	-	-	-	-	-	+	-	-	-	-	-
Aflatoxin B_1_	-	-	-	-	+/-	-	-	-	-	-	-	-	-	-	-	-	-	-	-
Aflatoxin B_2_	-	-	-	-	+/-	-	-	-	-	-	-	-	-	-	-	-	-	-	-
Aflatoxin M_1_	-	-	-	-	+/-	-	-	-	-	-	-	-	-	-	-	-	-	-	-
Aflatrem	-	-	-	-	+	-	-	-	-	-	-	-	-	-	-	-	-	-	-
Asparason A	-	-	-	-	+	-	-	-	+	-	-	-	-	-	-	-	-	-	-
Aspergamid A	-	-	-	-	-	-	-	-	-	-	+	-	-	-	-	-	-	-	-
Asperglaucide	-	+	-	-	-	-	-	-	-	-	-	-	-	-	-	-	-	-	-
Aspernigrin	-	-	-	-	-	-	-	+	-	-	-	-	-	-	-	-	-	-	-
Asperphenamate	+/-	-	-	-	-	-	-	-	-	-	-	-	-	-	-	-	-	-	-
Asperthecin	-	-	-	-	-	-	+	-	-	-	-	-	-	-	-	-	-	-	-
Aspertoxin	-	-	-	-	+/-	-	-	-	-	-	-	-	-	-	-	-	-	-	-
Aspochracin	-	-	-	-	-	-	-	-	-	+/-	-	-	-	-	-	-	-	-	-
Aspulvinone E	-	-	-	-	-	-	+	+	-	-	-	-	-	-	-	-	-	-	-
Aspulvinone O	-	-	-	-	-	-	+	-	-	-	-	-	-	-	-	-	-	-	-
Aurasperon B	-	-	-	-	-	-	-	+	-	-	-	-	-	-	-	-	-	-	-
Aurasperon C	-	-	-	-	-	-	-	+	-	-	-	-	-	-	-	-	-	-	-
Aurasperon G	-	-	-	-	-	-	-	+	-	-	-	-	-	-	-	-	-	-	-
Averantin	-	-	-	-	-	-	-	-	+	-	-	-	-	-	-	-	-	-	-
Averufanin	-	-	-	-	+	-	-	-	+	-	-	-	-	-	-	-	-	-	-
Averufin	-	-	-	-	+	-	-	-	+	-	-	-	-	-	-	-	-	-	-
Bilaid A	-	-	-	-	-	-	-	-	-	-	-	-	-	-	-	-	+	-	-
Butyrolactone I	-	-	-	-	-	-	+	-	-	-	-	-	-	-	-	-	-	-	-
Butyrolactone III	-	-	-	-	-	-	+	-	-	-	-	-	-	-	-	-	-	-	-
Butyrolactone V	-	-	-	-	-	-	+	-	-	-	-	-	-	-	-	-	-	-	-
Citreorosein	+	-	+	-	-	-	-	-	-	+/-	-	+	-	-	-	-	+	-	-
Citreoviridin	-	-	-	-	-	-	+	-	-	-	-	-	-	-	-	-	-	-	-
Citrinin	-	-	-	-	-	-	-	-	-	-	-	-	-	-	-	-	+	-	-
CNM 115,443	-	-	-	-	-	-	-	-	+	-	-	-	-	-	-	-	-	-	-
Cyclopiazonic acid	-	-	-	-	+	+	-	-	-	-	-	-	-	-	-	-	-	-	-
Demethylsulochrin	-	-	-	-	-	-	-	-	-	-	-	-	+	-	-	-	+	-	-
Deoxygerfelin	-	-	-	-	-	-	-	-	+	-	-	-	-	-	-	-	-	-	-
Diacetoxyscirpenol	-	-	-	-	-	-	-	-	-	-	-	-	-	+	-	-	-	-	-
Dihydrocitrinone	-	-	-	-	-	-	-	-	-	-	-	-	-	-	-	-	+	-	-
Dihydrosterigmatocystin	-	-	-	-	-	-	-	-	+	-	-	-	-	-	-	-	-	-	-
Dihydroxymellein	-	-	-	-	-	-	+	-	-	-	-	-	-	-	-	-	-	-	-
Emodin	+	-	+	-	-	-	+	-	+	+	-	+/-	+	-	-	-	+	-	-
Equisetin	-	-	-	-	-	-	-	-	-	-	-	-	-	+	-	-	-	-	-
Fallacinol	+	-	+	-	-	-	+	-	-	-	-	-	-	-	-	-	-	-	-
Flavoglaucin	+	-	+	+	-	-	-	-	-	-	-	-	-	-	-	-	-	-	-
Fonsecin	+	+	+	+	-	-	-	+	-	-	-	-	-	-	-	-	-	-	-
FR 901,235	-	-	-	-	-	-	-	+	-	-	-	-	-	-	-	-	-	-	-
Fumonisin B_2_	-	-	-	-	-	-	-	+/-	-	-	-	-	-	-	-	-	-	-	-
Fumonisin B_4_	-	-	-	-	-	-	-	+/-	-	-	-	-	-	-	-	-	-	-	-
Fusaric acid	-	-	-	-	-	-	-	-	-	-	-	-	-	-	-	+	-	-	-
Gibepyron D	-	-	-	-	-	-	-	-	-	-	-	-	-	-	+	+	-	-	-
Gliocladic acid	-	-	-	-	+	-	-	-	-	-	-	-	-	-	-	-	-	-	-
Glyantrypine	-	-	-	-	-	-	-	-	+	-	-	-	-	-	-	-	-	-	-
Heptelidic acid	-	-	-	-	+	-	-	-	-	-	-	-	-	-	-	-	-	-	-
Infectopyron	-	-	-	-	-	-	-	-	-	-	-	-	-	-	-	-	+	-	-
Isofusidienol	-	-	-	-	-	-	-	-	-	+/-	-	-	-	-	-	-	+	-	-
Iso-Rhodoptilometrin	+	-	+	-	-	-	-	-	-	+	-	+/-	+	-	-	-	+	-	-
Kojic acid	-	-	-	-	+	+	-	-	+	-	-	-	-	-	-	-	-	-	-
Kotanin A	-	-	-	-	+	-	-	+	-	-	-	-	-	-	-	-	-	-	-
Lecanoric acid	-	-	-	-	-	-	-	-	+	-	-	-	-	-	-	-	-	-	-
Malformin A	-	-	-	-	-	-	-	+	-	-	-	-	-	-	-	-	-	-	-
Malformin C	-	-	-	-	-	-	-	+	-	-	-	-	-	-	-	-	-	-	-
Mevastatin	-	-	-	-	-	-	+	-	-	-	-	-	-	-	-	-	-	-	-
Mevinolin	-	-	-	-	-	-	+	-	-	-	-	-	-	-	-	-	-	-	-
Monoacetoxyscirpenol	-	-	-	-	-	-	-	-	-	-	-	-	-	+	-	-	-	-	-
N-Benzoyl-Phenylalanine	+	+	-	-	-	-	-	-	-	-	-	-	-	-	-	-	-	-	-
Neocyclocitrinol	-	-	-	-	-	-	-	-	-	-	-	-	-	-	-	-	+	-	-
Neoechinulin A	+	-	+	+	-	-	-	-	-	-	-	-	-	-	-	-	-	-	-
Neoechinulin D	+	-	+	+	-	-	-	-	-	-	-	-	-	-	-	-	-	-	-
Neosolaniol	-	-	-	-	-	-	-	-	-	-	-	-	-	+	-	-	-	-	-
Nidurufin	-	-	-	-	+	-	-	-	+	-	-	-	-	-	-	-	-	-	-
Nigragillin	-	-	-	-	-	-	-	+	-	-	-	-	-	-	-	-	-	-	-
Norcitreoviridin	-	-	-	-	-	-	+	-	-	-	-	-	-	-	-	-	-	-	-
Norsolorinic acid	-	-	-	-	-	-	-	-	+	-	-	-	-	-	-	-	-	-	-
Notoamide Derivative	-	-	-	-	-	-	-	-	-	-	+	-	-	-	-	-	-	-	-
NP 15,352	-	-	-	-	-	-	-	-	-	+	+	-	-	-	-	-	-	-	-
*O*-Methylsterigmatocystin	-	-	-	-	+	-	-	-	-	-	-	-	-	-	-	-	-	-	-
Orsellinic acid	+	-	+/-	-	-	-	+	-	+	-	-	-	-	-	-	-	-	-	-
Oxalicine B	-	-	-	-	-	-	+	-	-	-	-	-	-	-	-	-	-	-	-
Paspalin	-	-	-	-	+	-	-	-	-	+/-	-	-	-	-	-	-	-	-	-
Paxillin	-	-	-	-	-	-	-	-	-	+	-	-	-	-	-	-	-	-	-
Penipurdin A	+	-	+	-	-	-	+	-	-	-	-	-	+	-	-	-	-	-	-
Physcion	+	-	+	-	-	-	-	-	-	-	-	-	+	-	-	-	-	-	-
Pinselin	-	-	-	-	-	-	-	-	-	-	-	-	-	-	-	-	+	-	-
Pyranonigrin	-	-	-	-	-	-	-	+	-	-	-	-	-	-	-	-	-	-	-
Pyrophen	-	-	-	-	-	-	-	+	-	-	-	-	-	-	-	-	-	-	-
Quinolactacin A	-	-	-	-	-	-	+	-	-	-	-	+	-	-	-	-	+	+	-
Quinolactacin B	-	-	-	-	-	-	-	-	-	-	-	-	-	-	-	-	+	-	-
Scalusamid A	-	-	-	-	-	-	-	-	-	-	-	-	-	-	-	-	+	-	-
Sclerotinin A	-	-	-	-	-	-	-	-	-	-	-	-	-	-	-	-	+	-	-
seco-Sterigmatocystin	-	-	-	-	-	-	-	-	+	-	-	-	-	-	-	-	-	-	-
Sterigmatocystin	-	-	-	-	+	-	-	-	+	-	-	-	-	-	-	-	-	-	-
Sydowinin A	-	-	-	-	-	-	-	-	-	-	-	-	-	-	-	-	+	-	-
Sydowinin B	-	-	-	-	-	-	-	-	-	-	-	-	-	-	-	-	+	-	-
Tensidol B	-	-	-	-	-	-	-	+	-	-	-	-	-	-	-	-	-	-	-
Terphenyllin	-	-	-	-	-	-	-	-	-	+	+	+	-	-	-	-	-	-	-
Terrein	-	-	-	-	-	-	+	-	-	-	-	-	-	-	-	-	-	-	-
Terrelulamide A	-	-	-	-	-	-	+	-	-	-	-	-	-	-	-	-	-	-	-
Triacetoxyscirpenol	-	-	-	-	-	-	-	-	-	-	-	-	-	+	-	-	-	-	-
Tryprostatin B	+/-	+	+	+	-	-	-	-	-	-	+	-	-	-	-	-	+	-	-
Tryptophol	-	-	+/-	-	-	-	-	+	-	-	-	-	-	-	-	+	-	-	+
Unugisin E	-	-	-	-	-	-	-	-	-	+	+	-	-	-	-	-	-	-	-
Ustiloxin B	-	-	-	-	+	-	-	-	-	-	-	-	-	-	-	-	-	-	-
Versicolorin A	-	-	-	-	+	-	-	-	+	-	-	-	-	-	-	-	-	-	-
Versicolorin C	-	-	-	-	+	-	-	-	+	-	-	-	-	-	-	-	-	-	-
Versiconal Acetate	-	-	-	-	-	-	-	-	+	-	-	-	-	-	-	-	-	-	-
Versiconol	-	-	-	-	+	-	-	-	+	-	-	-	-	-	-	-	-	-	-
Violaceol I	-	-	-	-	-	-	-	-	+	-	-	-	-	-	-	-	-	-	-
Violaceol II	-	-	-	-	-	-	-	+	+	-	-	-	-	-	-	-	-	-	-

The extrolite patterns of four xerophilic species in *Aspergillus* sections *Aspergillus* and *Restricti* were investigated (Table 2). Extrolite production was consistent in *A. chevalieri* (*n* = 2), *A. montevidensis* (*n* = 2) and *A. penicillioides* (*n* = 4), producing all the compounds detected in their cultures except for orsellinic acid and tryptophol which were produced only by two strains of *A. chevalieri*. Asperglaucide and asperphenamate were produced only by *A. penicillioides* and one *A. caesiellus* strain (DTO 463-A4), respectively. This finding agrees with the report that all species within the section *Restricti* produce any or both of the two compounds, whereas species in section *Aspergillus* do not make these compounds (Chen et al. 2017; Sklenář et al. 2017). N-Benzoyl-Phenylalanine was unique to both *A. caesiellus* and *A. penicillioides*, and could be a chemotaxonomic marker for these species and the section *Restricti*. *Aspergillus caesiellus* and *A. chevalieri* shared 11 extrolites; only citreorosein, emodin, echinulins, flavoglaucin, orsellinic acid and physcion have been reported in these species or within the two sister sections (Chen et al. 2017; Greco et al. 2015; Micheluz et al. 2016; Sklenář et al. 2017; Slack et al. 2009). In accordance with previous studies, the strains of *A. caesiellus*, *A. chevalieri* and *A. montevidensis* from the two sister sections shared production of neoechinulins A and D (Chen et al. 2017). Fonsecin and tryprostatin B were additionally detected in all species of the two sections but were not previously reported. The large number of metabolites shared among two sister sections confirms ancestral relationships of the species and sections, and emphasizes the importance of extrolite profiling in fungal taxonomy.

Within *Aspergillus* section *Flavi*, one strain was aflatoxigenic and three non-aflatoxigenic. The aflatoxigenic strain DTO-459-A2 produced B-aflatoxins, aflatoxin M_1_ and several aflatoxin biosynthetic precursors, but not 3-nitropropionic acid (3-NPA) (Table 2). Asparason A, gliocladic acid, heptelidic acid and ustiloxin B were found in cultures of all strains of *A. flavus*, confirming production by this species (Kovač et al. 2020; Umemura et al. 2014), while aspertoxin which was previously reported in *A. flavus* strains from Australia, Nigeria and USA (Uka et al. 2019) was produced only by the aflatoxigenic *A. flavus* strain DTO 459-A2 in the present study. The non-aflatoxigenic strains produced aflatrem, cyclopiazonic acid (CPA), kojic acid (KA) and paspalin, similar to the reports of Ezekiel et al. (2020b) for CPA and KA in non-aflatoxigenic *A. flavus* from dried ready-to-eat foods. The compounds 3-NPA, CPA and KA produced by *A. tamarii* were shared with *A. flavus* (Table 2).

The *A. versicolor* (section *Nidulantes*) strain examined in this study produced several extrolites similar to those from the section *Flavi* including asparason A, KA, very large amounts up to 59,000 µg/kg of the class 2B human carcinogen sterigmatocystin (STER) and derivative (seco-sterigmatocystin), and precursor compounds in the aflatoxin biosynthesis pathway (e.g., averantin, averufanin, averufin, dihydrosterigmatocystin, nidurufin, norsolorinic acid, versicolorins A and C, versiconal acetate and versiconol), but not aflatoxins (Table 2). This agrees with previous reports that this species produces STER (Jakšić Despot et al. 2017; Jurjević et al. 2013; Rank et al. 2011). The production of aflatoxin biosynthetic pathway metabolites has recently been reported in species within the series *Versicolores* of section *Nidulantes* to which *A. versicolor* belongs (Géry et al. 2023). Uncommon extrolites found in our strain included emodin, lecanoric acid, orsellinic acid, violaceol II, and possible signature extrolites such as CNM 115,443, deoxygerfelin, glyantrypine and violaceol I.

The examined *A. neoafricanus* strain (section *Terrei*) produced several extrolites including aspulvinone (E and O), butyrolactones (I, III and V), citreoviridin, mevinolin, terrein and traces of emodin that were previously reported in this species and the section *Terrei* (Barros Correia et al. 2020; Kiriyama et al. 1977; Samson et al. 2011b). A very large amount reaching 344,000 µg/kg of the neurotoxic citreoviridin was found in agar culture of the strain. Asperthecin, a compound common to section *Nidulantes* (Houbraken et al. 2020), as well as dihydroxymellein, mevastatin, norcitreoviridin, oxalicine B and terrelulamide A were reported as possible additional signature compounds in our *A. neoafricanus* strain. Fallacinol, large amount of orsellinic acid (> 130,00 µg/kg) and penipurdin A were also found in the agar cultures of *A. neoafricanus* (Table 2). The extrolite profiles of one strain (DTO 464-G3) of *A. wentii* (section *Cremei*) was examined in this study. Emodin, demethylsulochrin, iso-rhodoptilometrin and physcion were produced by this strain similar to previous reports for *A. wentii* in the literature (Houbraken et al. 2020). Additionally, 3-NPA and traces of penipurdin A were the uncommon extrolites in the agar culture of *A. wentii* (Table 2).

Three species (*A. subalbidus*, *A. neotritici* and *A. taichungensis*) in section *Candidi* were examined for extrolite production (Table 2). 3-hydroxyterphenyllin and terphenyllin were found in agar cultures of all the strains; these compounds are known to be produced by most species within the section *Candidi* (Kagiyama et al. 2016; Varga et al. 2007). *Aspergillus subalbidus* DTO 462-I3 strain produced aspochracin and traces of isofusidienol; these were not previously documented in this species. In addition, *A. subalbidus* DTO 464-E8 strain and both strains of *A. neotritici* produced citreorosein, whereas *A. neotritici* DTO 458-C4 strain and the two strains of *A. subalbidus* produced emodin and iso-rhodoptilometrin. Paspalin was found in agar culture of *(A) subalbidus* DTO 464-E8. *Aspergillus taichungensis* produced of tryprostatin (B) The signature compounds included aspergamid A and notamide derivative in *A. taichungensis*, NP15352 and unugisin E in *A. subalbidus* and *A. taichungensis*, and paxillin in *A. subalbidus*. The presence of notamide derivative but not taichunamides in *A. taichungensis* confirms the report of Kagiyama et al. (2016).

*Aspergillus niger* was the only species from the section *Nigri* examined in this study due to the high variability of extrolite production in this species (Frisvad et al. 2007a; Houbraken et al. 2020; Samson et al. 2007). For example, OTA was not detected in agar cultures of the six tested strains (Table 2), similar to previous reports from Nigeria (Akinfala et al. 2020; Ezekiel et al. 2020a), but other commonly reported compounds including aspernigrin, aspulvinone E, aurasperons, fonsecin, kotanin A, malformins, nigragillin, pyranonigrin, pyrophen and tensidol B were detected (Akinfala et al. 2020; Ezekiel et al. 2020a; Samson et al. 2007). *Aspergillus niger* DTO 458-G7 strain produced both fumonisins B_2_ and B_4_, distinguishing it from the other five strains, as well as large amounts of kotanin A. Although fumonisin B_2_, and B_4_, in rare instances, have been reported in *A. niger* strains from coffee beans, grapes and maize, or from strains intended for industrial applications (Biya 2022; Frisvad et al. 2007b, 2011; Han et al. 2017; Logrieco et al. 2009; Mogensen et al. 2010; Noonim et al. 2009), only FB_2_ was reported in strains from rice (Onami et al. 2018). Hence in the present study, FB_2_ and FB_4_ were co-produced by *A. niger* strains isolated from Nigerian rice for the first time. In addition, FR901235 was found in all strains and could possibly be an additional marker extrolite for this species.

Three *Fusarium* strains belonging to three species were examined and based on extrolite profiles, they were categorized into two. Group I consisted of strain DTO 458-B9 (= *F. duofalcatisporum*; *F. incarnatum-equiseti* species complex), which produced five type A trichothecenes (diacetoxyscirpenol, monoacetoxyscirpenol, triacetoxyscirpenol, 8-acetylneosolanol and neosolaniol) and equisetin. Group II strains (DTO 458-B5, *F. verticillioides* and DTO 458-E2, *F. youattiae*; both *F. fujikuroi* species complex) primarily produced gibepyron D in agar cultures (Table 2). The *F. youattiae* DTO 458-E2 strain also produced fusaric acid. It is important to note that *F. youattiae* was recently described from stem canker on *Mimosa pigra* in Australia (Tan et al. 2025), thus, we highlight the first extrolite pattern of this species. Overall, the extrolite patterns of all the *Fusarium* strains further support their taxonomic identifications in the species complexes (Ezekiel et al. 2020a; Janevska et al. 2016; Tan et al. 2025; Wang et al. 2023). Makun et al. (2011b) reported the presence of *F. chlamydosporum* and *F. verticillioides* in rice samples from Niger state, with the former producing DON and ZEN and the later FB_1_ and FB_2_. Although these toxins were not produced by the examined strains in the present study, the presence of *Fusarium* in the rice samples could pose some mycotoxigenic risks to consumers. The present study resents the first report of *F. duofalcatisporum* and *F. youattiae* in cereals in Nigeria.

We examined four *P. citrinum* strains and found several expected extrolites (Table 2). They include the nephrotoxic citrinin (CIT) and its metabolites dihydrocitrinone (DHC) and neocyclocitrinol, emodin and its derivatives citreorosein, iso-rhodoptilometrin, pinselin, scalusamid A, sydowinin A, and quinolactacins A and B in the agar cultures (Akinfala et al. 2020; Ezekiel et al. 2020b; Houbraken et al. 2011, 2020). Infectopyron, an *Alternaria* metabolite recently reported in *P. citrinum* strains from Nigeria (Akinfala et al. 2020; Ezekiel et al. 2020b), and tryprostatin B were also found in the cultures of all examined strains in the present study. The uncommon extrolites exclusively produced in *P. citrinum* strains were bilaid A, sclerotinin A and sydowinin B, which are reported here for the first time. Demethylsulochrin and isofusidienol were also found for the first time in *P. citrinum* cultures, although they were also produced by some Aspergilli. *Penicillium ezekielli* produced only quinolactacin A (Tan et al. 2022) while *W. mellicola* produced only tryptophol.

### Multi-mycotoxins in rice along the CPRP and IPRP chains

#### Mycotoxin co-occurrence in rice

**Fig. 5:**
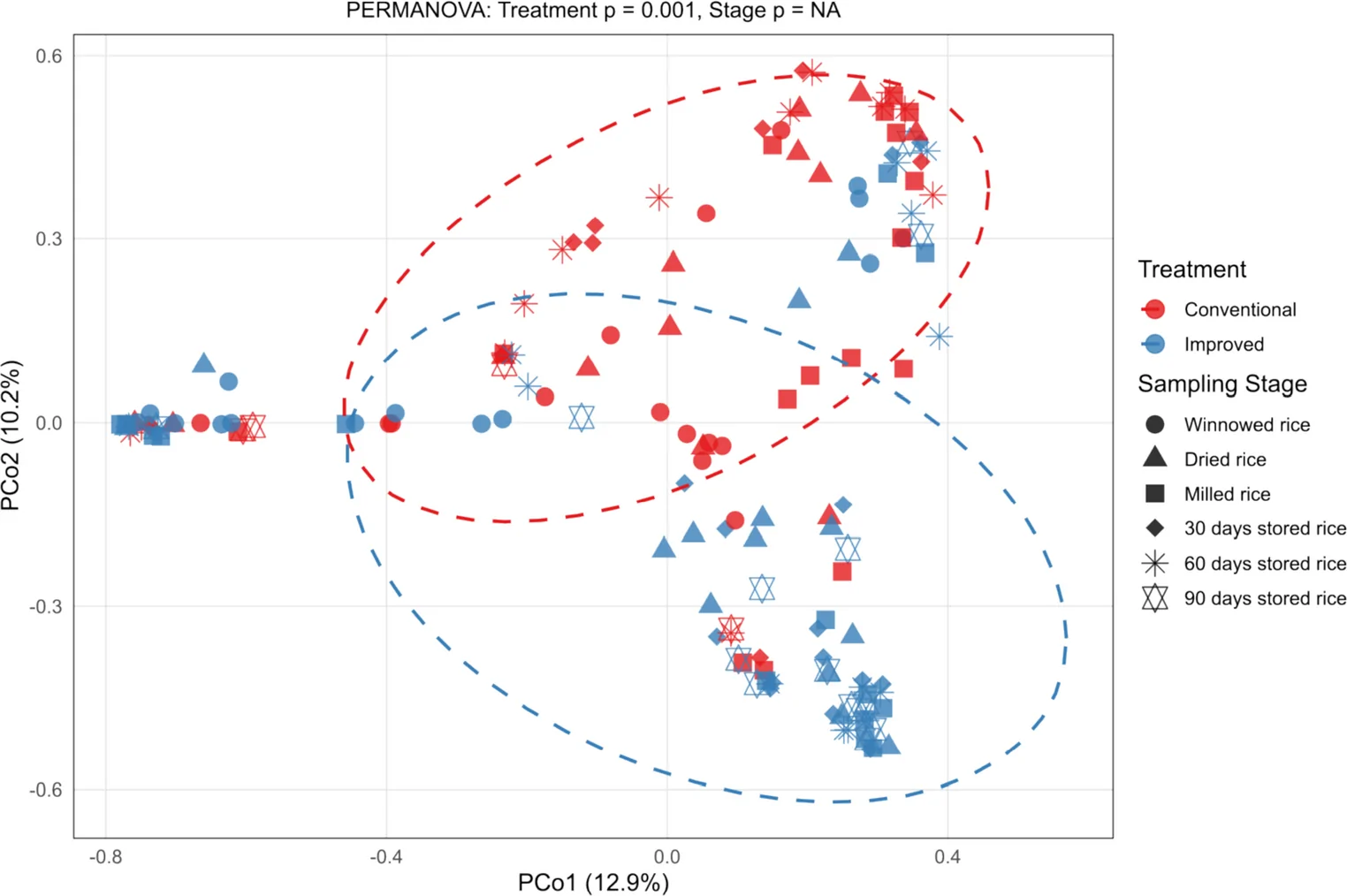
Principal Coordinates Analysis (PCoA) ordination plot of mycotoxin profiles in rice samples from the conventional and improved postharvest processing and storage chains. Different symbols represent sampling stages. Dashed ellipses indicate 95% confidence regions for each treatment group (PERMANOVA, *p* < 0.05)

The occurrence and levels of mycotoxins in food (e.g., rice) are important indices for measuring the effectiveness of food processing and storage conditions (Ayeni et al. 2021). In addition to mycotoxins occurring together with their producing fungi, they may also occur independent of mycotoxigenic fungal strains due to the stability of mycotoxins when the producing fungal strains are no longer viable (EFSA Panel on Contaminants in the Food Chain et al. 2020). Here, the presence of 15 mycotoxins classed into aflatoxins (AFB_1_, AFB_2_ and AFG_1_), citrinin (CIT and DHC), fumonisins (FB_1_, FB_2_ and FB_3_), zearalenones (ZEN and ZEN-sulfate), trichothecenes (nivalenol (NIV), and the emerging toxins (3-NPA, beauvericin (BEAU), MON and tenuazonic acid (TEN-AC) were monitored and detected in rice sampled along the two process chains including during storage. These mycotoxins co-occurred in the rice samples across the two process chains, albeit at varying proportions ranging from 3 to 6 classes. Principal Coordinates Analysis (PCoA) of the overall mycotoxin profiles confirmed a clear separation between rice samples from the two process chains (Fig. 5). Samples from the IPRP chain and CPRP chain formed partially overlapping but distinct clusters, suggesting that the postharvest processing method significantly shaped the overall mycotoxin community structure. The tighter clustering of IPRP samples in the lower region of the ordination space suggests greater homogeneity in mycotoxin profiles, consistent with the controlled mechanical drying and hermetic storage conditions of that chain. In contrast, the broader dispersion of CPRP samples reflects the higher variability in sun-drying and conventional jute bag storage conditions across farms and sampling stages.

Previous studies across Africa (Abdus-Salaam et al. 2015; Mahdjoubi et al. 2020; Makun et al. 2011a; Manizan et al. 2018; Onyedum et al. 2020), Asia (Eslamizad et al. 2021; Sinphithakkul et al. 2019; Siri-Anusornsak et al. 2022), and Europe (Juan et al. 2022; Monteiro et al. 2025) highlighted co-occurrence of mycotoxins in processed rice, which corresponds to milled or stored rice in the present study. Precisely, and similar to our findings, Makun et al. (2011a) detected 11 mycotoxins belonging to six classes: aflatoxins (AFB_1_, AFB_2_, AFG_1_ and AFG_2_), fumonisins (FB_1_ and FB_2_), ochratoxins (OTA), patulin, trichothecenes (DON and T-2 toxin), and ZEN in rice in Niger state, Nigeria. The co-occurrence of several mycotoxins in the rice samples at all stages of the process chains reiterates the involvement of diverse toxigenic fungi that invade rice, and presents the potential health risks consumers may face due to ingestion of rice contaminated with several mycotoxins. Mycotoxins of the various classes, identified in the rice samples, have been documented to have a link in stunting, neural tube defects, cancers (e.g., liver, kidney and esophageal) as well as organ and immune system toxicities, and estrogenic effects (Flajs and Peraica 2009; Missmer et al. 2006; Pestka 2010; Shirima et al. 2015; Sun et al. 2007; Warth et al. 2019).

#### Mycotoxin occurrence levels influenced by rice processing stages

The occurrences of mycotoxin classes in rice at the winnowing stages of the CPRP and IPRP chains ranged 46–100% and 57–100%, respectively, with aflatoxins and NIV having the lowest occurrences in winnowed rice in the respective chains (Table 3). In contrast, the occurrences of the mycotoxin classes in rice from the CPRP and IPRP chains after 90 days of storage in conventional jute bags and single-layer hermetic-type bags ranged 0–100% and 0–100%, respectively (Table 3). The higher occurrence of mycotoxins in winnowed rice from both process chains compared to other processing stages and in storage suggests that mycotoxin contamination of rice commenced primarily either on the field or during paddy harvesting activities (e.g., initial field drying and stacking/piling). However, the disparities in occurrences of the mycotoxins in the winnowed rice samples from the two process chains may have been due to several factors including variety of rice, distribution of fungi in the environment, rice cultivation and harvesting practices, and climatic conditions previously documented to influence mycotoxin contamination of crops globally (Hell and Mutegi 2011; Tang et al. 2019).

**Table 3:** Incidences of mycotoxin classes in rice across the stages of conventional and improved processing and storage chains

Mycotoxins	Occurrence (%) of mycotoxin classes										
	Conventional process and storage chain						Improved process and storage chain				
	Winnowed rice	Milled rice	30 days stored rice	60 days stored rice	90 days stored rice	Winnowed rice	Dried rice	Milled rice	30 days stored rice	60 days stored rice	90 days stored rice
Total aflatoxin	46.2	38.5	30.8	53.8	50.0	78.6	78.6	85.7	92.9	92.9	85.7
Total fumonisin	69.2	38.5	7.7	30.8	0.0	78.6	42.9	14.3	21.4	7.1	0.0
Total citrinin	84.6	46.2	38.5	38.5	25.0	71.4	71.4	78.6	71.4	71.4	92.9
Total zearalenone	92.3	100	92.3	92.3	75.0	100	92.9	85.7	85.7	92.9	85.7
Nivalenol	84.6	23.1	30.8	15.4	0.0	57.1	50.0	42.9	28.6	21.4	14.3
3-Nitropropionic acid	76.9	100	92.3	92.3	50.0	85.7	92.9	92.9	92.9	100	92.9
Beauvericin	100	100	100	100	100	100	100	100	100	100	100
Moniliformin	100	100	100	84.6	100	100	85.7	100	100	92.9	92.9
Tenuazonic acid	76.9	61.5	46.2	53.8	0.0	71.4	57.1	78.6	71.4	71.4	42.9

**Fig. 6:**
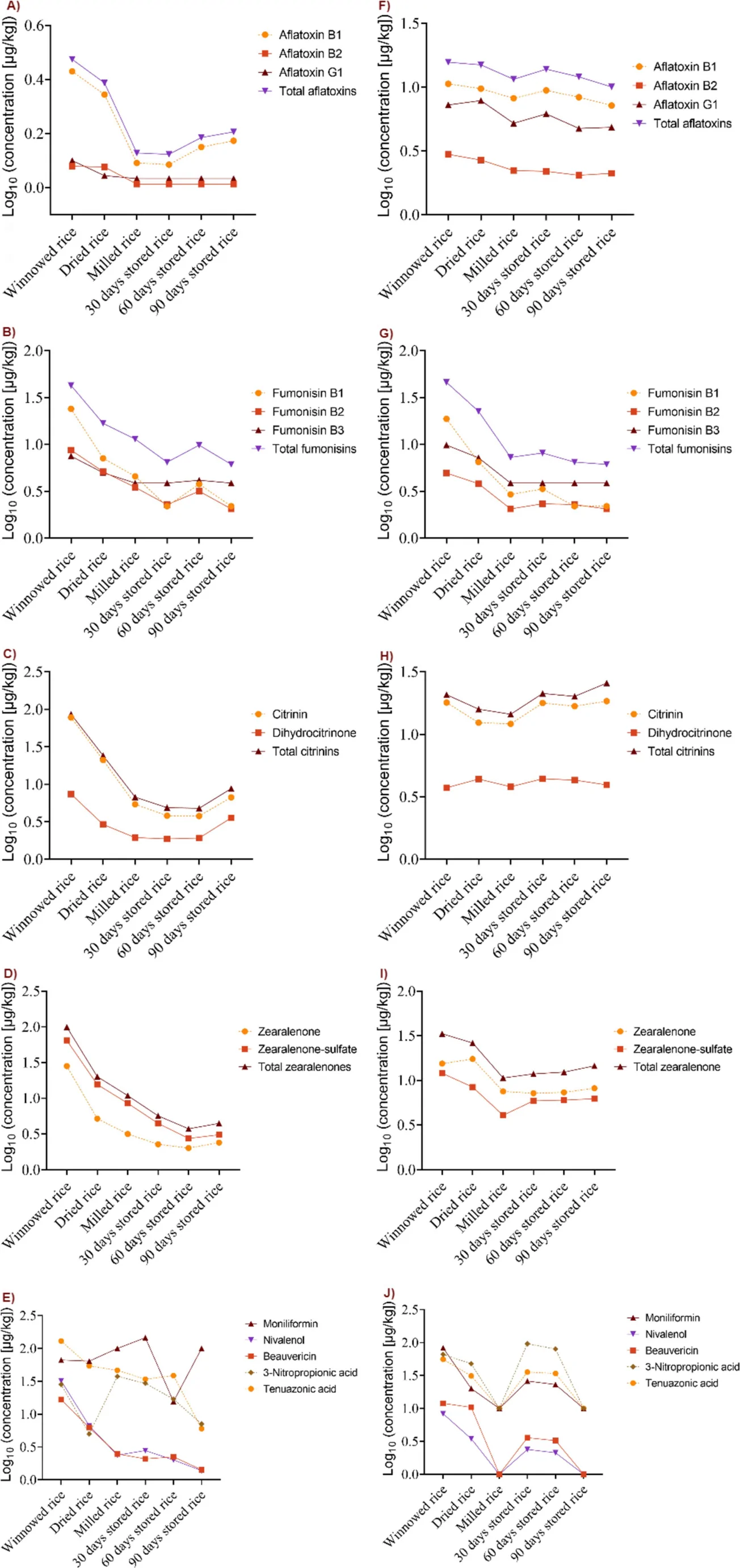
Mycotoxins in rice across the conventional and improved postharvest processing and storage chains. Panels A-E present the conventional postharvest processing and storage chain (CPRP) and panels F-J present the improved postharvest processing and storage chain (IPRP). Each horizontal pair shows the same mycotoxin class in the two chains: (A, F) aflatoxins; (B, G) fumonisins; (C, H) citrinins; (D, I) zearalenones; and (E, J) moniliformin, nivalenol, beauvericin, 3-nitropropionic acid and tenuazonic acid. Concentrations are expressed as Log_10_(1 + µg/kg)

With respect to mycotoxin levels in the rice along the two process chains, each analyzed sample was considered as a data point, such that < LOD values were replaced with LOD/2 for the mean calculations of all samples. This was necessary because some samples did not have detectable mycotoxin levels after 90 days of storage. The mean mycotoxin concentrations are presented here in µg/kg whereas as Log_10_(1 + µg/kg) to normalize the distribution of the concentrations in Fig. 6. The respective mean concentrations of AFB_1_ and total aflatoxins reduced from 4.17 to 4.77 µg/kg in winnowed rice to 0.25 and 0.36 µg/kg in milled rice prior to a steady increase in storage to 0.63 and 0.74 µg/kg after 90 days of storage under the CPRP chain (Fig. 6A). In contrast in the IPRP chain, the respective mean levels of AFB_1_ and total aflatoxins drastically reduced from 135 to 250 µg/kg in winnowed rice to 29.3 and 53.9 µg/kg in milled rice and then slightly to 26.7 and 49.1 µg/kg after 90 days of storage in single-layer hermetic-type bags resulting in 8.9% reduction of aflatoxins in storage (Fig. 6F). There was drastic reduction in mean concentrations of FB_1_ and total fumonisins in the CPRP chain from 89.5 to 158 µg/kg in winnowed rice to 1.20 and 5.60 µg/kg in 30 days stored rice, which remained same after 90 days of storage in conventional jute bags resulting in 85% and 69% reductions in storage, respectively (Fig. 6B). Similarly, the mean levels of FB_1_ and total fumonisins drastically reduced in the IPRP chain from 36.3 to 1690 µg/kg in winnowed rice to 3.27 and 7.21 µg/kg in milled rice and further to 1.20 and 5.14 µg/kg after 90 days of storage in single-layer hermetic-type bags resulting in 63 and 29% reduction in storage, respectively (Fig. 6G). Total CIT levels in the CPRP chain reduced from 1121 µg/kg in winnowed rice to 64.8 µg/kg in milled rice but increased to 208 µg/kg after 90 days of storage (Fig. 6C). Similarly, in the IPRP chain, there was a drastic reduction of total CIT levels from 204 µg/kg in winnowed rice to 61.6 µg/kg in milled rice and then an increase in storage to 225 µg/kg after 90 days of storage in single-layer hermetic-type bags (Fig. 6H). The respective mean concentrations of total ZEN, BEAU, NIV, and TEN-AC in winnowed rice (362, 34.9, 76 and 598 µg/kg) from the CPRP chain also reduced to 16.9, 1.97, 3.70 and 175 µg/kg in milled rice and further to 5.32, 0.46, 0.39 and 5.00 µg/kg in rice after 90 days storage in conventional jute bags with a marked 69, 77, 90 and 97% reduction in storage (Fig. 6D, I, E and J). For the IPRP chain, total ZEN reduced from 74.8 µg/kg in winnowed rice to 20.3 µg/kg in milled rice and then increased gradually in storage up to 37.7 µg/kg after 90 days of storage in single-layer hermetic-type bags (Fig. 6D and I), whereas storage of rice in single-layer hermetic-type bags for 90 days resulted in percentage reduction levels of 7, 20, 22 and 40% for 3-NPA, MON, TEN-AC and NIV from initial concentrations of 500, 57, 74.4 and 3.1 µg/kg in milled rice, respectively (Fig. 6E and J).

Based on the mycotoxin concentration data recorded for rice samples from the two process chains, it is obvious that processing and storage conditions played crucial roles in the extent of mycotoxin contamination of the samples; this has been documented for several crops (Fandohan et al. 2006; Matumba et al. 2015; Sudini et al. 2015; Tang et al. 2019; Walker et al. 2018). For example, mycotoxin concentrations were generally reduced in rice processed between the winnowing and milling stages in the two chains, and these lay claim to the impact of soaking and floatation washing on mycotoxin decontamination of grains (e.g., maize and rice) (Canela et al. 1996; Matumba et al. 2015; Van der Westhuizen et al. 2011). To be specific, the floatation technology for decontamination of grains contaminated with aflatoxins was long patented (Hagen et al. 1989), and the applied technology subsequently removed at least 11–17% of fumonisins from maize in a study in South Africa (Van der Westhuizen et al. 2011). Additionally, the duration of grain soaking may have influenced the consistent decontamination recorded between the winnowing and milling stages of the CPRP chain compared to the IPRP chain. Prolonged soaking of grains initiates fermentation, which tilts microbial diversity in favor of fermenting communities (e.g., yeasts, and bacteria though not included in this study). Fermentative microbes, including yeasts, are known to possess the capacity to degrade, absorb or convert mycotoxins to less toxic forms (Banwo et al. 2023; Ezekiel et al. 2025). Although the impact of fermentation microbes on mycotoxin reduction in rice is yet to be documented, yeasts have been reported in soaking/fermentation processes of rice (Adeniran et al. 2012; Ramalingam and Anthoni Raj 1996). Our findings of certain species in rice from the CPRP chain agree with previously reported yeasts in rice and they include those of technological importance in food fermentation (e.g., *Meyerozyma* (Liu et al. 2020), *Pichia* (Adeniran et al. 2012), *Rhodosporidiobolus* (Xinwei et al. 2021), *Saccharomycopsis* (Cai et al. 2019), and *Wickerhamomyces* (Mohammadi Shad and Atungulu 2020). The aforementioned species were most likely instrumental in decontaminating the grain of mycotoxins in the present study; validating this hypothesis requires further extensive studies. Adopting a 4–7-day soaking duration for rice grains prior to drying in the IPRP chain could be a credible post-harvest mycotoxin control step in the rice value chain.

The drying conditions applied in the two process chains seemed to be effective to reduce mycotoxin levels in the rice. This agrees with the acceptable moisture contents of the dried rice detailed in previous sections above. Milling/dehusking of rice in the two process chains was deemed fit to control mycotoxin production, except for 3-NPA and MON in both process chains. The reasons for the observed disparity may be that: (a) toxigenic fungal propagules responsible for 3-NPA and MON production were located within the inner parts of the pericarp and/or endosperm and preferred production of the toxins in the inner grain parts between the drying and milling steps to spike the toxin levels in the milled rice; or (b) mycotoxins were more concentrated in the endosperm which the dehusking process exposed. Either of the views may not be conclusive and requires further investigations. However, it is known that mycotoxins are usually concentrated in the grain seed coats, which makes de-coating, dehulling or dehusking an effective step for mycotoxin removal when the seed endosperm is to be consumed (Afolabi et al. 2015; Okeke et al. 2018). But, the efficacy of this process in mycotoxin decontamination depends on fungal diversity and load, as well as on the extent of mycotoxin transfer into the grains (Fandohan et al. 2006; Njapau et al. 1998). Our findings for elevated 3-NPA and MON levels in milled rice from the two process chains seem to agree with the report of Matumba et al. (2015), where significant higher proportions of NIV, DON, ∑AcDON (acetyl-deoxynivalenol; type B trichothecenes) were observed in maize endosperm after dehulling compared with other mycotoxins. In line with their suggestion and reports from Schwake-Anduschus et al. (2014), moisture levels may have affected dissolution and diffusion of 3-NPA and MON during production to the inner parts of the rice grain.

It is known that longer periods of grain storage especially under poor conditions elevate mould and aflatoxin incidences (Kaaya and Kyamuhangire 2006). The aforementioned fact together with optimal oxygen levels may have accounted for the consistent rise in aflatoxin levels in rice stored in the conventional bags across the 90-day storage period. The most critical condition for aflatoxin production is sufficient aeration, which is common when conventional jute bags are used for storage (Hesseltine et al. 1966). With respect to storage, a general outlook of the data indicated that there was consistent control (reduction or relative stability) of the mycotoxins during storage of the rice in single-layer hermetic-type bags in the IPRP chain compared to storage in conventional jute bags in the CPRP chain, although the data were not statistically significant. For example, the relatively lower total aflatoxins, total fumonisins, 3-NPA, MON, NIV and TEN-AC levels in rice after 90 days of storage in single-layer air-tight bags utilized in this study compared to the levels in milled rice at the onset of storage in the IPRP chain attest to the efficient mycotoxin control potential in rice of the hermetic-type bags. For some of the mycotoxins (e.g., total aflatoxins, CIT and MON), storage in conventional bags resulted in relatively increased levels after 90 days storage. Our findings are similar to those of Murashiki et al. (2018), Ng’ang’a et al. (2016), Sudini et al. (2015), and Walker et al. (2018) where grain storage in hermetic-type bags or silos prevented spikes in aflatoxin and/or fumonisin levels compared to levels documented for grains from traditional storage devices. Ng’ang’a et al. (2016) demonstrated that after 35 weeks of maize storage in hermetic/PICS bags at an initial moisture content < 14%, total aflatoxin levels did not change compared to the 5–8 folds increase in conventional (polypropylene and jute) bags. Murashiki et al. (2018) reported as much as 95 times higher aflatoxin levels in maize stored in conventional bags compared to hermetic bags. They also documented no significant effect of storage type (conventional vs. hermetic bags) on fumonisin levels in stored maize grains. Similarly, Walker et al. (2018) reported no difference in fumonisin levels in conventional and hermetic bags after six months of storage. Although no significant differences were observed for total aflatoxin levels between milled rice and 90 days stored rice in each of the storage conditions, there were marked differences which agree with previous reports. Hermetic storage of grains suffocates aerobic fungal propagules by limiting atmospheric gaseous exchange leading to almost static levels of mycotoxins in storage (Diarra and Amoah 2019; Hossain et al. 2019; Sudini et al. 2015; Walker et al. 2018; Williams et al. 2017b). Besides aflatoxins and fumonisins, which have been studied with respect to grain storage in conventional and hermetic bags, the dynamics of CIT, NIV, ZEN, 3-NPA, BEAU and TEN-AC in grains under hermetic storage is reported here for the first time.

A unique pattern was observed for total CIT where the concentrations of this nephrotoxin in rice stored in either of the conventional and single-layer hermetic-type bags increased progressively until the end of the 90 days storage period. This finding suggests that total CIT production was unaffected by oxygen and CO_2_ tension, which obviously encouraged optimal biosynthesis of the nephrotoxins. Hasan (1993) reported that stress-induced CO_2_ evolution led to a rise in respiratory activity and CIT synthesis when 0.1% camphore was supplemented in fungal culture, while Pereira et al. (2008) reported that CIT production was a function of dissolved oxygen concentration in batch cultures. Hossain et al. (2019) reported that oxygen concentration of rice paddy dropped from 25% to < 5% within 29 days of storage in hermetic bags. Similarly, Kalsa et al. (2019) reported oxygen concentration drop from 20% to 17.88%, 15.36%, and 10.22% after storage of wheat in Super GrainPro bags for two, four and six months, respectively. Thus, total CIT may have been influenced by the involvement of oxygen availability together with other nutritive elements (e.g., fatty acids, nitrogen and carbon sources) and environmental factors including water activity, temperature and storage conditions, which were previously suggested as regulators of CIT production (Kamle et al. 2022). Nevertheless, it is necessary that further investigations into the exact respiration (oxygen and CO_2_) requirements for CIT production in stored grains are conducted.

For total ZEN, the pattern observed during rice storage in conventional bags, where there was reduction compared to storage in single-layer hermetic bags where the concentrations of the estrogenic mycotoxin increased, may have been mediated by competition of toxigenic strains with toxin-degrading fungi (and bacteria though not studied here) in favour of the latter group. Several reports are available to support possible decontamination potentials of anaerobic (facultative/obligate) bacteria against some of the investigated mycotoxins which reduced during rice storage in any of the process chains (Ferrara et al. 2021; Ndiaye et al. 2022). This idea is suggested because ZEN production is greatly influenced by oxygen concentration (Greenhalgh et al. 1983) and the conventional bags had sufficient access for environmental oxygen exchange. Conversely, the increase recorded total ZEN levels during rice storage in single-layer hermetic-type bags was minimal and is supported by literature that 20–60% CO_2_ and 5–20% oxygen levels, which is lower than environmental levels were capable of limiting ZEN biosynthesis in corn (Paster et al. 1991).

## Conclusion

This study has provided a comprehensive insight into the distribution and toxigenicity of fungi in rice along two postharvest process chains into storage, and the influence of the process chains and storage bag types on fungal and multiple mycotoxin contamination of rice. Diverse genera and species of fungi including toxigenic mould strains, and industrially valuable yeast and mould species, were identified in rice from the two processing chains. Rice from the CPRP chain had higher incidences of toxigenic moulds in storage compared to rice stored in the single-layer hermetic-type bags. The rice from both process chains contained several mycotoxins, which were reduced at varying levels depending on the process stage and storage bag type. The use of single-layer hermetic bag, which may be more affordable than multiple layer hermetic bags, generally limited fungal growth and mycotoxins in storage, although the jute bags seemed to favour reduction of certain mycotoxins. The present study is limited by inability to commence rice sampling from the field through processing to storage. The inclusion of an extended (e.g., 7-day) soaking duration for rice grains in the IPRP chain may offer the required optimized post-harvest mycotoxin control step in the rice value chain. In addition, further areas of research related to profiling the functional microbial communities in local rice processing as an option to determine and exploit their mycotoxin reduction potentials is proposed as a future perspective. Furthermore, there is an urgent research need to explore the optimization of standard postharvest rice process and storage conditions for the subsistence farmer in Nigeria, which can serve as a model for other countries in Africa.

## Data Availability

Data generated in this study may be available on reasonable request.
